# How to Validate *in silico* Deployment of Coronary Stents: Strategies and Limitations in the Choice of Comparator

**DOI:** 10.3389/fmedt.2021.702656

**Published:** 2021-08-17

**Authors:** Francesca Berti, Luca Antonini, Gianluca Poletti, Constantino Fiuza, Ted J. Vaughan, Francesco Migliavacca, Lorenza Petrini, Giancarlo Pennati

**Affiliations:** ^1^Laboratory of Biological Structure Mechanics, Department of Chemistry, Materials and Chemical Engineering “Giulio Natta”, Politecnico di Milano, Milan, Italy; ^2^Biomechanics Research Center (BioMEC), Biomedical Engineering, School of Engineering, College of Science and Engineering, National University of Ireland Galway, Galway, Ireland; ^3^Department of Civil and Environmental Engineering, Politecnico di Milano, Milan, Italy

**Keywords:** arterial stenting procedure, finite element analysis, *in vitro* test, mock-up vessel, 3D printing

## Abstract

This study aims at proposing and discussing useful indications to all those who need to validate a numerical model of coronary stent deployment. The proof of the reliability of a numerical model is becoming of paramount importance in the era of *in silico* trials. Recently, the ASME V&V Standard Committee for medical devices prepared the V&V 40 standard document that provides a framework that guides users in establishing and assessing the relevance and adequacy of verification and validation activities performed for proving the credibility of models. To the knowledge of the authors, only a few examples of the application of the V&V 40 framework to medical devices are available in the literature, but none about stents. Specifically, in this study, the authors wish to emphasize the choice of a relevant set of experimental activities to provide data for the validation of computational models aiming to predict coronary stent deployment. Attention is focused on the use of *ad hoc* 3D-printed mock vessels in the validation plan, which could allow evaluating aspects of clinical relevance in a representative but controlled environment.

## Introduction

Computational models have been used for years in support of the design and testing phases of medical devices, without clear indications on how to assess the relevance and adequacy of verification and validation (V&V) activities performed for proving the credibility of models. Recently, the ASME V&V Standard Committee for medical devices prepared a document that is intended to fill this gap, providing the so-called Risk-Informed Credibility Assessment Framework, known briefly as V&V 40. The framework is based on the concept that the use of each model in decision-making for a medical device is associated with an intrinsic risk, which may cause undesirable impacts. There could be minor to significant consequences according to the involvement of the simulation outputs in the decision-making process (1).

In brief, the path indicated by V&V 40 foresees the following steps: (i) statement of the question of interest, containing the specific decision or concern that is being addressed; (ii) definition of the Context of Use (COU), which specifies the role and scope of the computational model in addressing the question of interest; (iii) assessment of the model risk within the COU and the potential consequences of an incorrect decision, generally evaluated in terms of harm on the patient health; (iv) establishment of the verification, validation, and applicability activities and the related goal for the credibility factors, meaning the determination of the rigor needed for each step of the V&V activities such that the model credibility is commensurate with the model risk; (v) planning of the activities to prove credibility, namely, the tests allowing the evaluation of the model results against a comparator study and, after the execution of such plan, (vi) model assessment, whose result might require a revision of the whole path.

Among these steps, the definition of the question of interest and, consequently, the COU are the crucial aspects to account in the model preparation, while, at the same time, the choice of the comparator study, namely, the set of data against which the model is evaluated at the validation step, has major effects in the final assessment of model credibility. As far as the authors know, only few examples of the application of the V&V 40 framework are available in the literature (2–4), but none about cardiovascular devices, and, in particular, stents for the treatment of coronary atherosclerosis.

Coronary atherosclerosis is a complex and multifactorial disease responsible for the development of abnormalities in a vessel wall, among which stenosis due to the growth of atherosclerotic plaque. The common treatment to restore a stenotic lumen is percutaneous coronary intervention (PCI), which involves the implantation of a coronary stent (5). Over time, these medical devices experienced great technological innovation up to their establishment as the golden standard for obstructive atherosclerotic vascular disease (6, 7).

The preparation and use of the numerical model of a stent represent a multi-step activity, since they have to take into account (i) the device production phase, namely the crimping of the stent on the balloon and catheter to obtain the delivery system, which affects the mechanical behavior and ii) the interaction with the human body that starts with the implantation inside the body. Particularly, in this second case, the definition of the validation activities becomes strategic.

The occasion for approaching and developing this theme was given by the European project InSilc, devoted to the development of an *in-silico* platform for the prediction of coronary stent implantation performance in individual cardiovascular physiology. In this study, the authors, once they have introduced the appropriate question of interest and COU for the setup of the computational model of coronary stent deployment, will discuss in detail the choice of a relevant comparator for the validation plan according to the V&V pathway.

A good comparator should provide the most suitable support to the use of the computational model for the COU, meaning to identify those tests allowing equivalency in the type and range of inputs and outputs between the model and the experiment. The better this correspondence, the higher the credibility level achievable by the model.

In this sense, *in vivo* cases of deployment provide the best degree of realism, but the access to quantitative data is limited, commonly based only on images, and the level of uncertainties affecting these measurements is extremely high. These uncertainties are even more prominent in the numerical model of the vessel, whereby the geometry is reconstructed based on clinical images, and the material properties are taken from the literature, as it is not possible to perform a real-time and *in-situ* characterization. Another solution involves the use of data from deployments in animals: this can provide more accessible data from a living environment (i.e., *ex vivo*) but is not representative of the lesion complexity of a human case. A well-controlled environment for V&V activities can be obtained performing *in vitro* deployments, where the delivery system is released in a mock vessel, where the geometrical features and material properties are known. In this way, it is also possible to have good input data for setting up the virtual model; clearly, a higher level of accuracy is expected. This approach was followed in (8), where a cylindrical PVC conduit was used, and in (9) where straight elastomeric tubes were adopted.

However, to increase the level of realism of *in vitro* tests, more sophisticated mock vessels are required: a realistic geometry should account for tortuosity and lumen variability (due to the presence of stenosis), and the mechanical properties of the wall should be representative of the compliance of the vessel. This would enable validation of the deployment process through *in vitro* tests that evaluate aspects of clinical relevance in a representative but controlled environment. Clearly, it is necessary to find a compromise between the controllability and realism of the experiment selected for the assessment of numerical results. A possible strategy for achieving this appropriate balance could be the use of 3D-printed vessels: this would allow developing *ad hoc* arteries made of materials that can be subjected to mechanical characterization tests.

In this context, once the question of interest and COU have been defined, this study will propose an affordable comparator study involving *in vitro* tests and discuss its use in the validation process, aiming to provide useful indications to all those who need to validate a numerical model describing the deployment procedure of coronary stents in virtual patients and its effects under acute conditions (i.e., stent-vessel interaction).

## Materials and Methods

**Figure 1:**
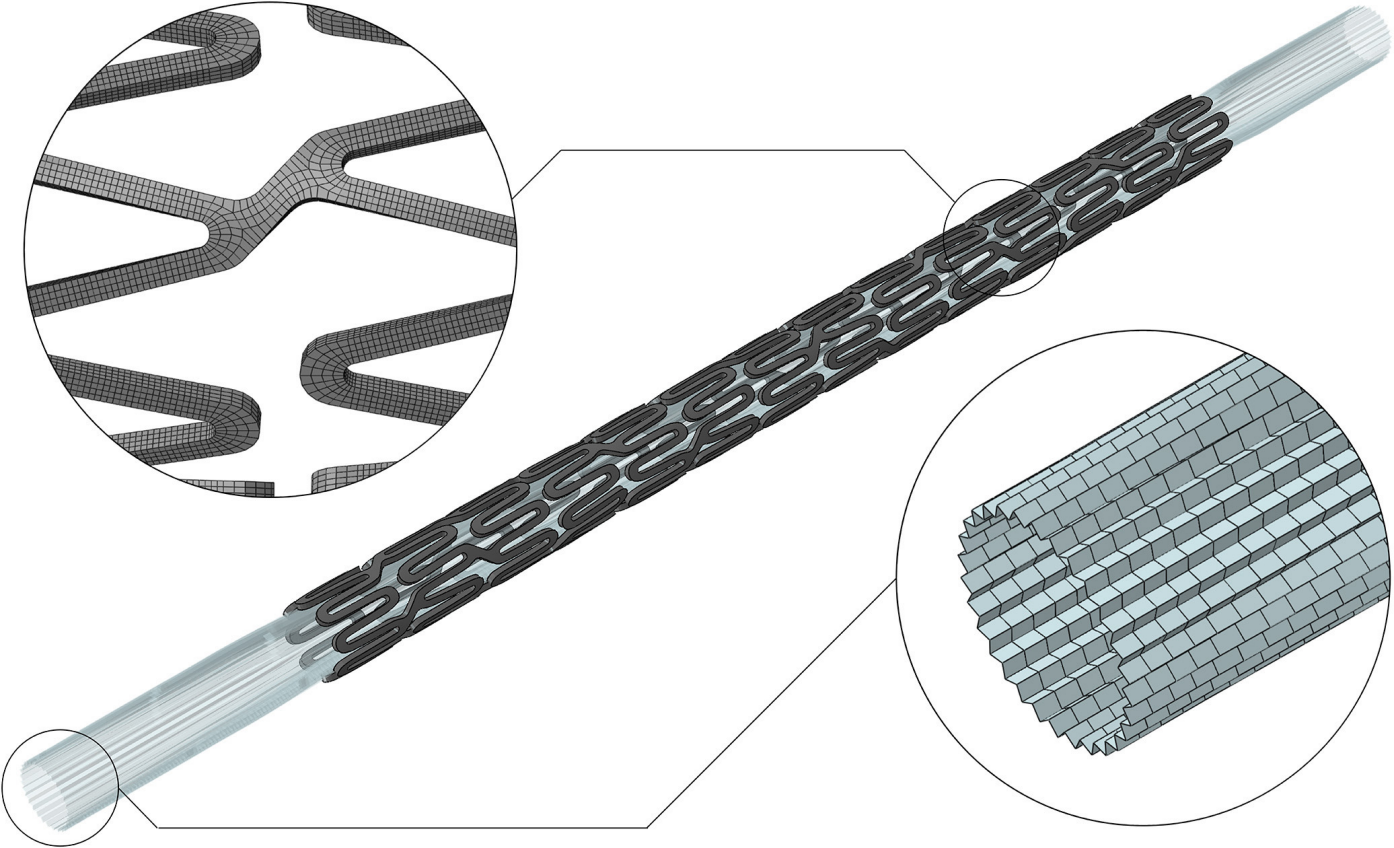
Digital twin of the SYNERGY™ BP delivery system, with a detail of the mesh refinement of the stent and balloon (8).

As a starting point for the development of an implantable device numerical model able to describe complex interactions, it is required to perform single-component validation (10). In the case of the stent delivery system, it entails accurately knowing the device geometry, the constituent material, and performing a systematic comparison between the model outputs and some selected data from tests representative of the intended use. In this study, the SYNERGY™ BP (Bioabsorbable Polymer) Everolimus-Eluting Platinum Chromium Coronary Stent, was used and provided by Boston Scientific Limited (BSL, Boston, MA, United States). Indeed, BSL was a partner in the InSilc project and made available to the authors few samples of the complete delivery system. The corresponding computational model used in this study includes the stent and the balloon, and is reported in Figure 1. Thanks to the information on the geometric characteristics and material properties provided by the manufacturers, the stent model was developed. A mesh of 177,312 hexahedral elements with reduced integration (C3D8R) was selected after a thorough mesh sensitivity analysis. For the balloon, the model proposed in the study of Chiastra et al. (11) was adopted, using 14,520-membrane elements (M3D4). Finally, a validation activity, based on simple *in vitro* tests, was performed. The description of the *in silico* model development and validation are reported in a recent study of the authors (8).

Once an accurate model of the device is available, it is possible to proceed with the application of the V&V pathway to a coronary stent deployment computational model, which requires the statement of the question of interest. From a clinical point of view, it may be formulated as “is the stent able to properly restore the physiological lumen when implanted into a diseased artery?”. From an *in silico* perspective, it can be specifically interpreted as:

i) What is the inner diameter of the vessel after stent implantation?

Indeed, the post-treatment diameter measurement provides information on residual stenosis;

However, other implications related to the question of interest might be:

ii) What is the diameter reached by the artery during the maximum inflation of the balloon? This measure, which indicates the maximum stretch reached by the vessel wall, is associated with the risk of damage and consequently the risk of inducing restenosis;
iii) Has any strut malapposition been found?

Fluid dynamics disturbed by struts not perfectly apposed is associated with an increased risk of thrombosis.

The consequent COU refers to the use of the numerical model of a coronary stent delivery system for the evaluation of the performance of the stent during the acute phase of the treatment. In particular, since this stage of the procedure is dominated by the mechanical interaction between stent radial stiffness and vessel compliance, the model could provide an assessment of the quantities defined by the question of interest. At this stage, following the V&V guidelines, the user should discuss the model risk to properly establish the rigor needed to evaluate the validation activities (i.e., quantification of the uncertainties). However, it is not in the scope of this study to perform a step-by-step risk-informed credibility assessment. Instead, as already stated, this study aims to discuss possible choices and criticalities in the definition of the comparator study and its use for validating the *in silico* model. In the next sections, the setup and the execution of the *in vitro* and corresponding *in silico* tests are described. All the simulations were performed in the Abaqus 2019 environment (Dassault Systemes Simulia Corp., Johnston RI, United States).

### Mock Vessels

According to the COU, for the validation plan, it was decided to perform *in vitro* deployment tests into 3D-printed mock vessels, since in the acute phase of the stenting treatment no biological response has to be described. Different vessel morphologies, from simplified ones to realistic cases, were considered for the phantoms.

The choice of the constituent material for manufacturing phantom vessels represents an important aspect. Indeed, the *in vitro* deployments should occur in tubular vessels characterized by small dimensions, able to create stent-vessel interactions resembling the *in vivo* conditions, and, at the same time, must withstand the balloon inflation up to very high pressures (12–14 atm). Moreover, material stiffness should be in the *in vivo* range for coronary arteries. In the frame of the InSilc project, the authors had access to the PolyJet technology, which is reported as one of the standards in the literature of 3D-printing of anatomical phantoms because of its high resolution (12). This would allow mimicking the dimensions, shape, and all morphological aspects (e.g., stenoses) affecting the outcome of the deployment in terms of lumen gain and malappositions. Among the printable materials, the Agilus30 polymer was recognized as the most suitable material for producing mock arteries (13). Although its mechanical properties are available in the manufacturer data sheet (14), it was preferred to perform characterization tests on material samples for proper calibration of the computational material model.

#### Material Characterization

**Figure 2:**
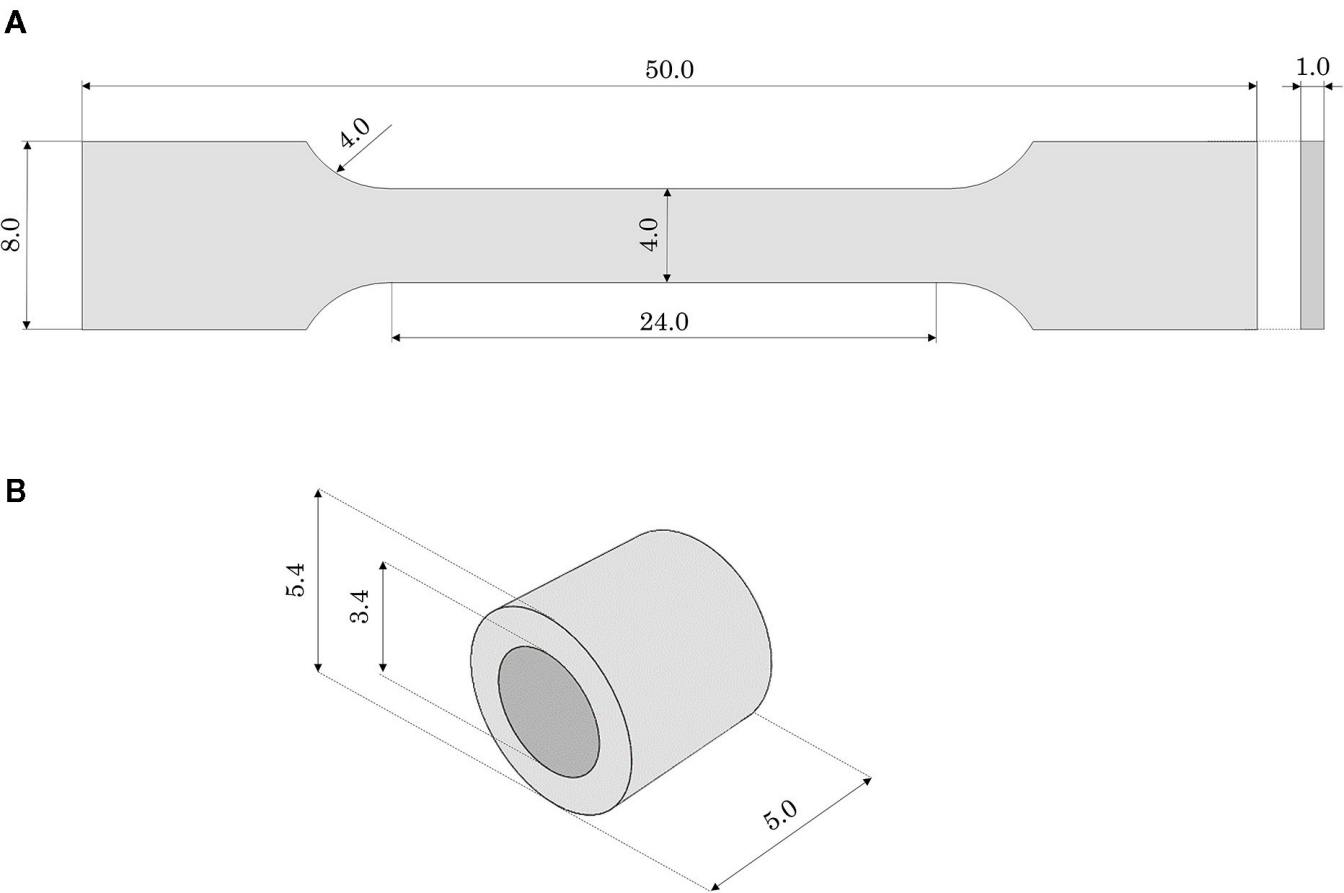
Geometrical features (in mm) of **(A)** the dog-bone and **(B)** the ring samples made of Agilus30.

Dog-bone samples can be used for mock vessel material tensile behavior characterization. In this work, dog-bone samples of Agilus30 were produced: the gauge length (*L*_0_) was 24 mm, with a rectangular cross-section of 4 × 1 mm thickness (Figure 2A). Since the performances of the dog-bone sample could be affected by the orientation of the samples in the 3D printer, the samples were printed aligned with the printing plane both with longitudinal (L) and transverse (T) orientations to the print direction. Experimental tests were performed with the samples immersed in water and at a controlled temperature comparable with body temperature (37 ± 1°C). Uniaxial tensile tests were conducted using MTS Synergie 200 H (MTS Systems, Eden Prairie, MN, United States), mounted with a 100 N load cell at 3.36 mm/s (15). A first test was carried out until the failure of the specimen to evaluate the ultimate strain limit. Subsequent tests were conducted up to lower strain values (assessed 110% true strain, corresponding to a crosshead displacement of 48 mm): this was due to the choice of adding a holding phase of 40 s at the end of the loading phase to assess material relaxation. The unloading process was performed at the same velocity.

Because of specimen sizes and the testing environment, the use of optical methods or extensometers for the measurements of the strains in the gauge length was unsuitable. Accordingly, the material model properties were identified reproducing the tests *in silico* and calibrating the values to match the force-displacement *in vitro* results. These simulations utilized an implicit solver.

The computer-aided design (CAD) model of the dog-bone sample was discretized using hexahedral solid elements with reduced and hybrid formulation and hourglass control (C3D8RH). The material response was modeled with a hyperelastic law through a first-order Neo-Hookean strain energy potential, assuming an incompressible material:


(1) $$\begin{array}{r} U=C_{10}\left(\bar{I_{1}}-3\right) \end{array}$$

where *U* is the strain energy potential, $\bar{I_{1}}$ is the first deviatoric strain invariant, and *C*_10_ is a material parameter to be calibrated over the experimental curves.

As a further step in the material characterization, annular tensile tests, with a loading phase, followed by holding and unloading phases, were performed. With these tests, it was possible to investigate the material performance related to the quality of the bond between successive layers in the printing process. For this purpose, annular samples (external diameter 5.4 mm, inner diameter 3.4 mm, total length 5 mm) were printed in the horizontal position (Figure 2B). Moreover, this testing configuration would allow a deformation mode better mimicking the use of the phantom during stent deployment (namely radially loaded by the stent expansion). *Ad hoc* steel pins allowed to test the ring specimens applying an internal displacement: the samples were tested with the same testing machine, under the same environmental conditions, and applying the same strain rate as the dog-bone tensile test (immersed in water at 37 ± 1°C). Before running the tests, using the material model properties identified with the dog-bone tensile test simulation and assuming cylindrical pins as rigid surfaces, a numerical simulation of the test on a ring sample was performed: it allowed to assess the displacement boundary condition to be applied in the experiment to reach the strain same as in the tensile test (namely 110% true strain). Accordingly, the tests were initially performed imposing a loading phase up to a crosshead displacement of 12 mm (110% maximum true strain). Unfortunately, in the first two tests, specimen failure occurred before reaching the end of the loading phase. Hence, other experiments were run up to a lower value (crosshead displacement up to 4 mm) that could guarantee the integrity of the specimen during the whole test (namely loading, holding, and unloading). These experiments were replicated numerically and the force-displacement outputs were compared.

#### Vessel Design

**Figure 3:**
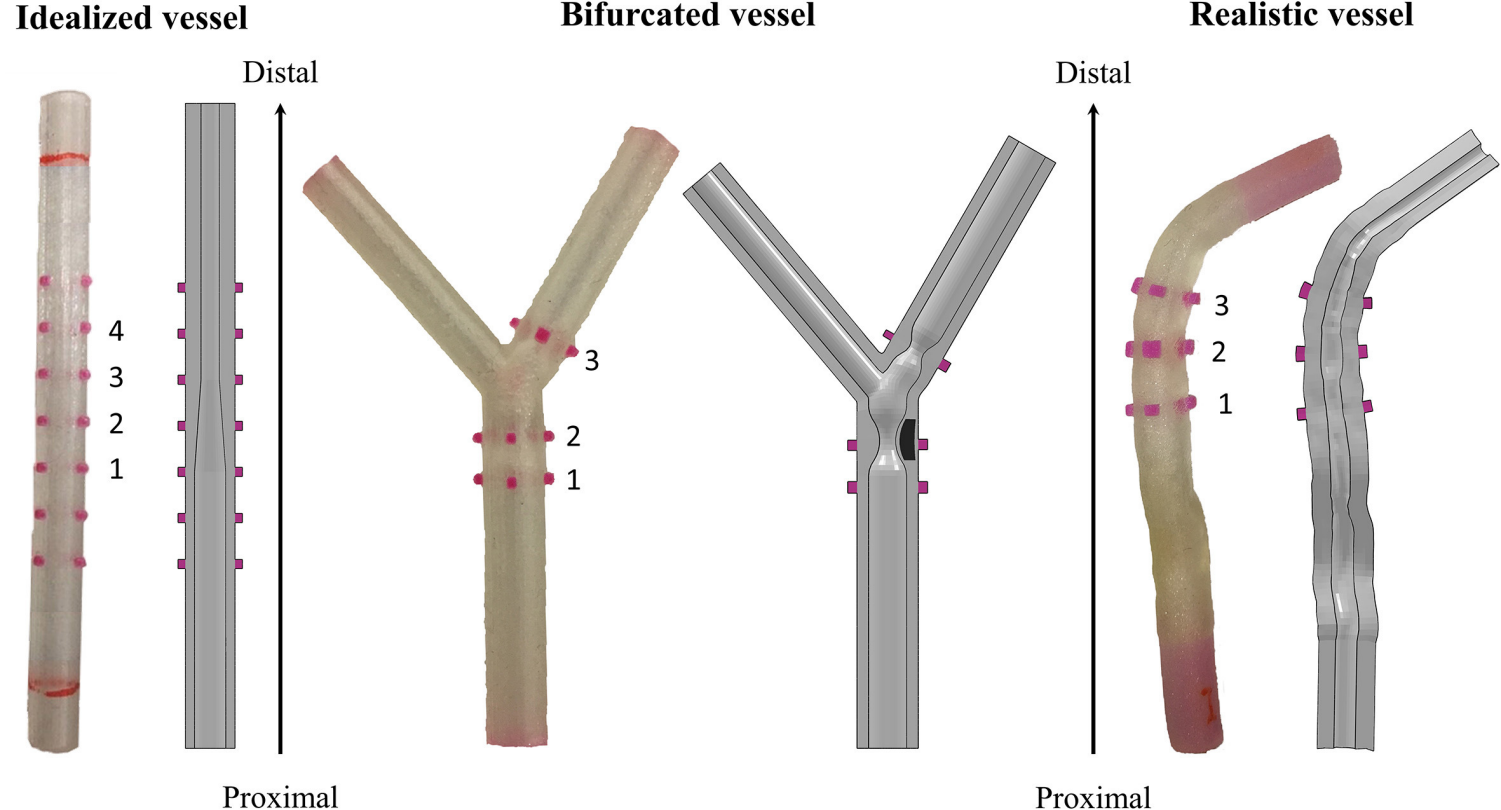
Different designs of the 3D-printed mock vessels with an indication of the markers ID for the comparison between experiments and simulations. Each of the realistic phantoms is reported on the left of the corresponding CAD, which is shown as a section to highlight the morphology of the lumen.

Three different designs of mock vessels were considered in the study, presented from the most simplified to the more complex (Figure 3):

- idealized straight vessels with variable lumen, whose dimensions were chosen because of similarity to those of human coronaries. The outer diameter was maintained constant at 5.4 mm, while the internal diameter varied from 2 to 3.4 mm. The whole sample measured 50 mm in length. The narrow part of the vessel mimicked the presence of a stenosis, while the larger diameter was chosen to potentially investigate the situation in which some of the stent struts are malapposed;
- bifurcated vessels with a rigid component in the lumen mimicking calcifications. Although the bifurcation design was idealized, the proportions between the main branch, proximal and distal portions, and the side branch were inspired by realistic measures (16). The level of realism of this case was increased by the presence of two localized stenoses, among which one was made of a rigid material deposited during the printing phase. This represents a good testing condition since the stent, which has to be implanted across the bifurcation, is required to interact with portion of a vessel characterized by different types of compliance. Indeed, one part of the stent would interact with the rigid component mimicking the calcification, and another would experience an almost free expansion because of the presence of the ostium (i.e., the void in the wall due to the side branch);
- realistic geometries reconstructed from optical coherence tomography (OCT) images and processed through a preliminary simulation. In fact, before stent implantation, balloon angioplasty is an established procedure for guaranteeing both a safe delivery system insertion and a satisfactory lumen expansion, and increasing the success of stenting. Since the mock vessels are made of an elastomeric material, with mostly an elastic behavior, they cannot mimic the response of the plaque to the angioplasty (i.e., the fact that the lumen remains open when the balloon deflates). For this reason, it was decided to perform a preliminary numerical simulation of angioplasty to widen the lumen at the stenosis and then print this deformed configuration for performing the experimental stent deployment.

The final printed product was semi-transparent, allowing the location of the deployed stent to be monitored but precluding the direct measure of the lumen and/or the stent features. For this reason, and following the previous literature on stent deployments (8, 9), and to answer the question of interest, it was decided to select the outer diameter of the mock vessels as the measurements of interest in the comparator study. These are simple data to be acquired through a commercially available optical system.

However, it could be not trivial to trace a definitive correspondence between the measurements acquired on the real specimen and those obtained from the *in silico* simulations during the assessment phase. Hence, for guaranteeing an equivalency between the model output and the experimental data, all the mock vessels had rigid colored markers added to serve as a reference for the measurements. The markers were placed in two opposed pairs (the diameters identified by each pair are perpendicular to each other) in significant positions depending on the geometry of the considered vessel. The idealized vessel was equipped with seven sets of markers to cover its whole length, while bifurcated and realistic ones had only three because of the more defined location for the implantation (inspired by the clinical case).

CAD models of the three designs were prepared and used for both producing the phantoms and preparing the numerical simulations (Figure 3). Once printed, a preliminary inspection was performed for evaluating the discrepancy between the CAD dimensions and those of the real phantom. Since all the deployment tests would be performed in a wet environment at 37 ± 1°C to mimic the body environment, this assessment was performed with the vessels immersed in temperature-controlled water. In this way, the same conditions of the characterization tests on the material samples were maintained. A high-resolution professional camera was used for the image acquisition (Canon EOS 6D, with a sensor CMOS with 20.2 megapixels and dimensions 36 × 24 mm; the lens is a macro Canon MP-E 65 mm f/2.8 1-5 × with fixed focus–focal distance: 65 mm, Canon, Tokyo, Japan). Each image had one-vessel geometry, with a focus on the markers, and was analyzed using the ImageJ software (National Institutes of Health, Bethesda, MD, United States) to evaluate the measures of the diameters under the initial condition. The value of the outer diameter was measured at the edges of the markers (both the proximal and distal ones), each of which was labeled with a progressive number from the proximal to the distal portion of the vessel (Figure 3). To account for inter/intra-user variability, the diameter values were obtained as an average of six values taken independently by two operators.

For preparing numerical models, the CADs of mock vessels were discretized with hexahedral elements with reduced integration (C3D8R), with a number of elements ranging between 22,360 and 34,068. The material response was described with the calibrated Neo-Hookean material model. The markers and the inclusion of the bifurcated vessel, being printed with a material much stiffer than Agilus30, were assumed perfectly rigid, and modeled as non-deformable parts.

### Stent Deployment

#### *In vitro* Test

Experimental deployment tests were performed using coronary SYNERGY™ BP delivery systems (3 mm of nominal diameter and 16 mm of length) (BSL, Boston, MA, United States). To mimic the implanted environment, all deployment tests were performed using the previous experimental setup that allowed to keep the specimen immersed in water at a controlled temperature of 37 ± 1°C for the preliminary inspection. The same high-resolution camera was used to capture the test, namely, to monitor the increase in vessel outer diameter due to stent expansion.

To prevent translations and rotations of the specimens, all the mock vessels were constrained at their ends to rigid supports, which also maintained the accessibility of the lumen for delivery system insertion.

Once the delivery system was inserted inside the vessel, the desired position was adjusted on the observation of a colored marker on the catheter. The stent was deployed by pressurizing the balloon to the desired pressure with a manual inflator.

All the experiments were performed with a constant rate (1 atm/s) both during the loading and unloading phases. In the tests involving idealized vessels, the balloon was inflated to a final pressure of 11 atm; in the bifurcated vessels the inflation reached a value of 8 atm, while in the realistic case the maximum pressure reached 14 atm, according to the suggested clinical procedure.

To allow the acquisition of measurements at several time-steps, the operator maneuvering the inflator declared with his/her voice the progressive change in the pressure value. In this way, during the post-processing phase, it was possible to evaluate the quantities of interest in the video frames corresponding to each pressure increment of one atmosphere.

This enabled the generation of a diameter-pressure curve that helps in the interpretation of the results, if compared with the only outputs of interest cited in the description of the question of interest, namely, at the maximum inflation and the end of the test.

The post-processing phase followed the same procedure described for the measurement of the diameters of the initial vessels. Each frame was analyzed using the ImageJ software to evaluate the measures of the outer diameters at the proximal and distal edges of each marker, at targeted pressure values. To account for inter/intra-user variability, the diameter values were obtained as an average of six values taken independently by two operators.

In the case of the idealized vessels, as a further method of investigation, micro-computed tomography (μCT) imaging of the vessel with the inserted stent was performed at the end of the deployment. This allowed acquiring additional information, which cannot be evaluated in real-time with the available experimental setup, such as the final stent configuration or possible presence of strut malapposition.

#### Numerical Simulation

The experiments were replicated numerically using an explicit solver, providing a quasi-static regimen. All the simulations consisted of multiple steps:

- Positioning: the stent-balloon system, as shown in Figure 1, (8) was moved to fit the deployment site. For simulations of stent deployment in the idealized vessel, whose lumen was straight, it was sufficient to position the device according to the experimental procedure, whereas for the bifurcation and realistic vessel an *ad hoc* strategy was adopted. The stent and balloon were deformed using an external cylinder that was moved in displacement control mode for the cylinder axes to coincide with the centerline of the mock vessel. This strategy allowed to obtain a simplified positioning step that was representative of the correspondent experiment in the case of scarce curvatures of the considered vessels. The boundary conditions to be applied to each of the cylinder nodes were computed with an external Matlab script (MATLAB 2018b, The MathWorks, Inc., Natick, MA, United States). During this step, the following contacts pairs were activated: cylinder-stent, cylinder-balloon, balloon-stent, stent-self, and balloon-stent.
- Inflation: a progressively increasing uniform pressure was applied on the internal surface of the balloon, and the maximum value was reached during the experimental activity. The load history followed a sigmoidal pattern over the step time to avoid abrupt pressure changes that could compromise the quasi-static regime of the simulation. During this phase, the displacements of the balloon extremities were constrained to reproduce the balloon fixation on the catheter. To account for the interactions between different parts, balloon-stent, stent-artery, balloon-artery, stent-self, and balloon-self contacts were activated.
- Deflation: the pressure was reduced progressively down to a slightly negative value to obtain balloon deflation and to allow reaching the elastic equilibrium between the mock vessel and the stent. The same contact pairs of the previous step were activated during the deflation phase.

A comparison between the model and experimental outputs was performed in terms of clinically relevant quantities, i.e., vessel diameter at maximum inflation and vessel diameter gain (defined as the difference between the final vessel diameter and the initial one, normalized over the initial one). In the case of the idealized vessel, an evaluation of the vessel lumen and stent configuration was provided in comparison with the μCT images.

## Results

In the next sections, the comparison of the *in vitro* tests and corresponding *in silico* results is described. For experimental deployments, the results of a single representative test for each configuration were reported and used as a basis for discussion, with no aim of addressing any credibility assessment that would have required uncertainty quantification based on repeated tests.

### Mock Vessels

#### Material Characterization

**Figure 4:**
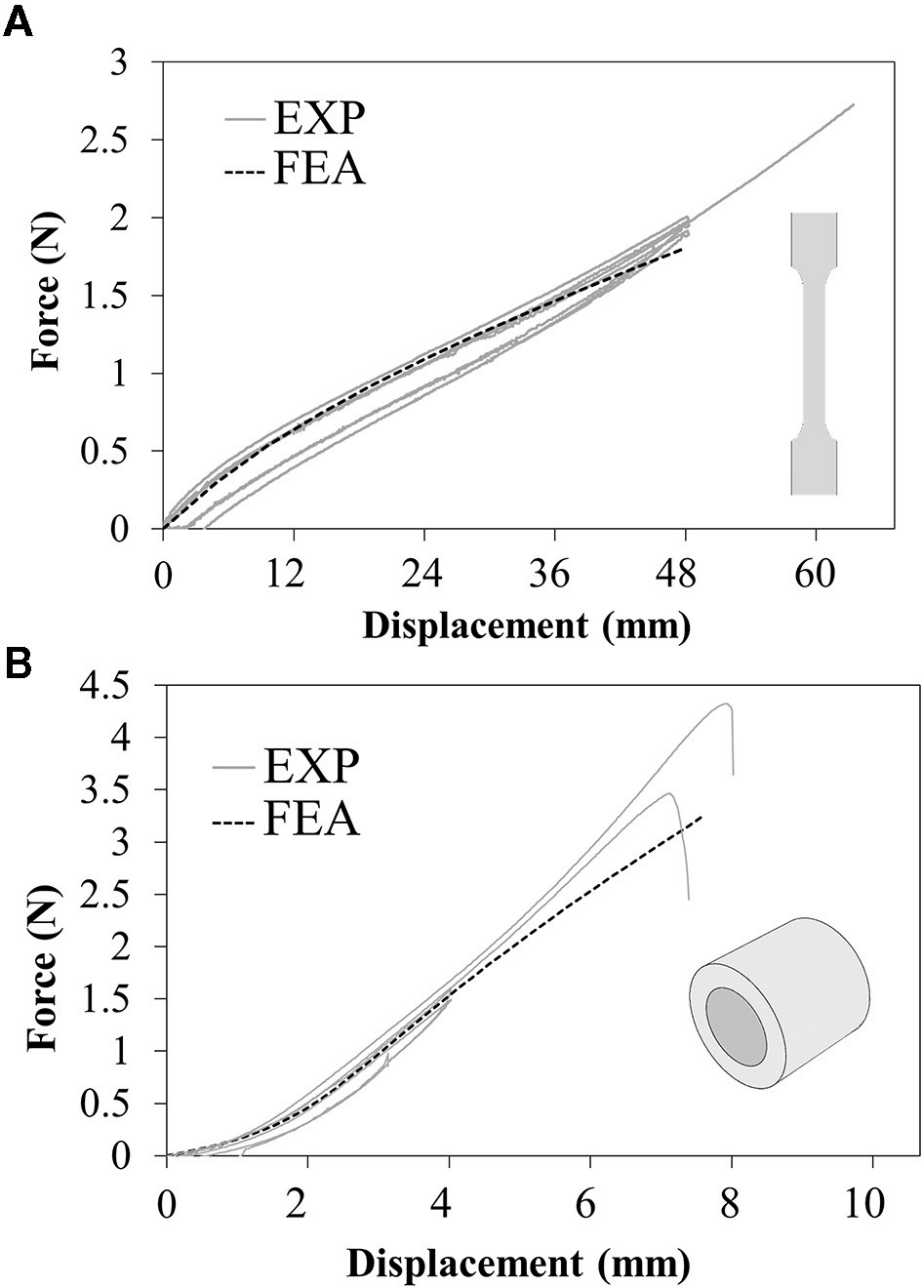
Force-displacement plots resulting from experimental tests (solid lines) and numerical simulations (dashed lines) of the **(A)** uniaxial tensile tests on dog-bone samples and **(B)** annular tensile tests on ring samples.

The results of tensile tests on the dog-bone specimens showed that different in-plane printing orientations (L or T) did not affect remarkably the tensile mechanical properties (Figure 4A). Moreover, during the holding phase, no significant relaxation and a very small hysteresis at the unloading was detected, allowing to confirm the assumption of neglecting viscous phenomena. According to these data, the values of *C*_10_was calibrated in numerical simulations equal to 97 KPa. The numerical model was used to assess the ultimate strain value at 130% (corresponding to the applied crosshead displacement of 65 mm). A very good match was obtained up to 40 mm displacement, with subsequent softening of the numerical plot compared with the experiments. This could be explained by the choice of a first-order Neo-Hookean law, which might be less accurate when dealing with very high deformations (Figure 4A).

The annular tests showed a strong reduction in ultimate strain compared with the previous tests, specifically corresponding to a local strain value between 71 and 79% estimated through the numerical model (Figure 4B).

#### Vessel Design

All the vessel designs were successfully printed, and the mismatch between the CAD dimensions of the outer diameter and the real values (in a wet environment at body temperature) stayed in the range of ±3%, meaning up to a difference of 190 μm that corresponds to double the stent strut thickness (detailed values are summarized in Table 1).

**Table 1:** Comparison between the initial value of the diameter in the CAD and the real phantom of the idealized, bifurcated, and realistic vessel design.

**Marker**	**Initial idealized vessel diameter (mm)**				**% Err**	
**ID**	**CAD**		**EXP (mean value)**			
	**Proximal**	**Distal**	**Proximal**	**Distal**	**Proximal**	**Distal**
1	5.40	5.40	5.56	5.59	3.0%	3.5%
2	5.40	5.40	5.58	5.55	3.3%	2.8%
3	5.40	5.40	5.53	5.51	2.4%	2.0%
4	5.40	5.40	5.53	5.56	2.4%	3.0%
**Marker**	**Initial bifurcated vessel diameter (mm)**				**% Err**	
**ID**	**CAD**		**EXP (mean value)**			
	**Proximal**	**Distal**	**Proximal**	**Distal**	**Proximal**	**Distal**
1	5.29	5.29	5.31	5.32	0.4%	0.6%
2	5.29	5.29	5.27	5.28	−0.4%	−0.2%
3	4.70	4.70	4.74	4.79	0.9%	1.9%
**Marker**	**Initial realistic vessel diameter (mm)**				**% Err**	
**ID**	**CAD**		**EXP (mean value)**			
	**Proximal**	**Distal**	**Proximal**	**Distal**	**Proximal**	**Distal**
1	5.39	5.26	5.22	5.23	−3.2%	−0.6%
2	5.00	4.89	4.90	4.87	−2.0%	−0.4%
3	5.24	4.84	5.16	4.77	−1.5%	−1.4%

### Stent Deployment

The outputs of deployment tests in terms of vessel diameter-pressure relationship can be divided into loading and unloading phases: the lower portion of the curve represents the loading phase, characterized by an almost constant diameter (from 0 to about 3.5–4 atm) that rapidly increases in the dynamic phase of balloon expansion (up to 5.5 atm) and proceeds with a less steep gain until maximum inflation pressure; then, the upper part of the curve represents the unloading phase, where the pressure is lowered and the diameter progressively reduces its value.

**Figure 5:**
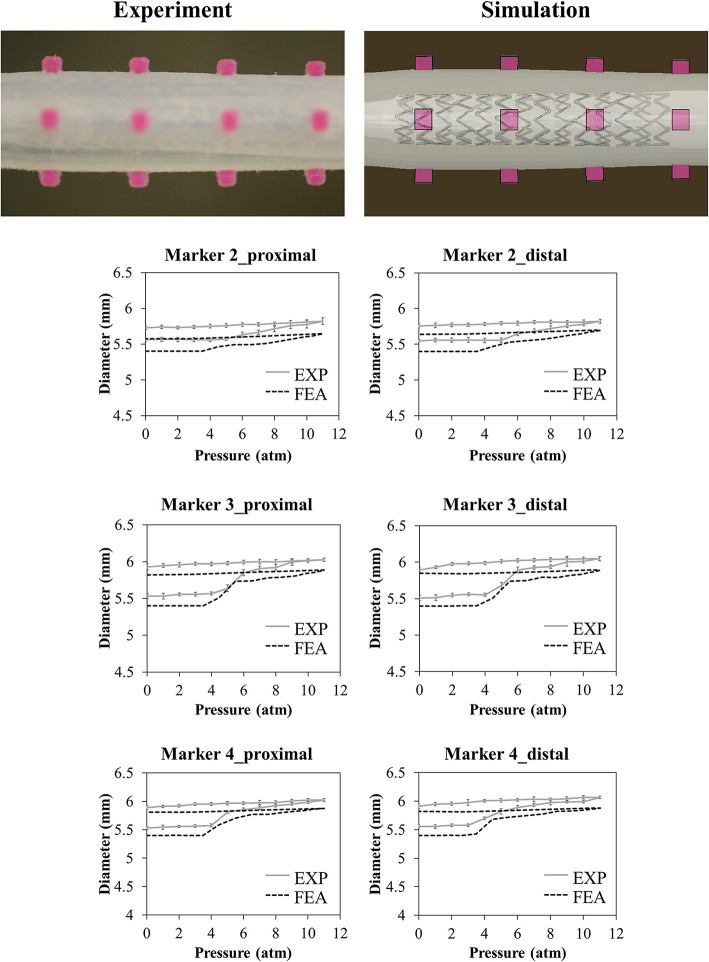
Comparison between experimental (mean values and relative experimental variability) and numerical results of the deployment of SYNERGY™ in an idealized vessel, taken at Marker 2, 3, 4 (from proximal to distal, Marker 1 remained unaffected by the procedure due to its lateral position). A qualitative comparison between experimental and numerical configuration is reported considering the final instant of the balloon deflation phase.

The results of the deployment in the idealized vessel are reported in Figure 5. Since Marker 1 did not show any experimental change in its position because of pressure variation (its position was in the area of wider diameter where a complete malapposition was expected), the corresponding plots were not shown. The choice of evaluating many pressure levels could identify where the model and the experiments exhibited poor match and evaluate the reasons behind that. Indeed, the curves obtained by the model seemed almost a direct translation of the experimental ones, proving that the model was able to capture the experimental data of outer diameter gain at each marker with a good agreement (Table 2). The initial mismatch of diameter (at a pressure equal to 0 atm) did not depend on the simulation, but it was related to the already observed geometrical differences (Table 1) between the CAD and the mock vessel.

**Table 2:** Comparison between the diameter gain obtained in the numerical simulations and experiments involving the idealized vessel design.

	**Vessel diameter gain (%)**			
**Marker**	**FEA**		**EXP (mean value)**	
**ID**	**Proximal**	**Distal**	**Proximal**	**Distal**
2	3.3%	4.4%	2.7%	3.8%
3	7.8%	8.3%	7.2%	6.9%
4	7.6%	7.8%	6.5%	6.3%

**Figure 6:**
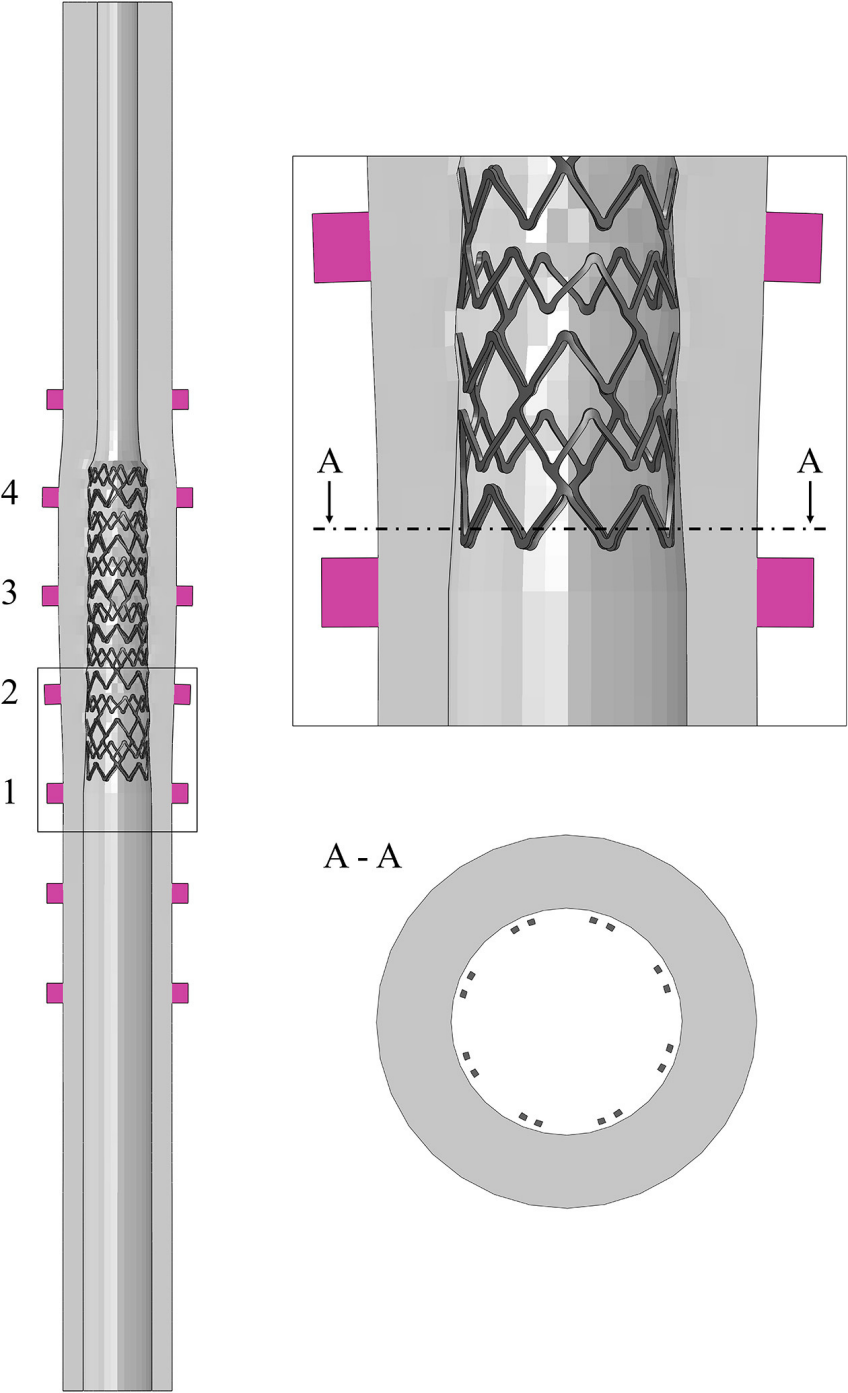
Simulation of the deployment in the idealized vessel, with a detail of the struts malapposition in correspondence to Marker 1.

The numerical model was used to investigate the stent behavior inside the vessel, with a perfect apposition in the distal portion characterized by the narrow lumen (Markers 2, 3, and 4) and strut malappositions in the area characterized by a wider lumen next to Marker 1 (Figure 6). From the simulation, it was possible to measure the lumen gain in the stenotic portion of the vessel of about 40%.

**Figure 7:**
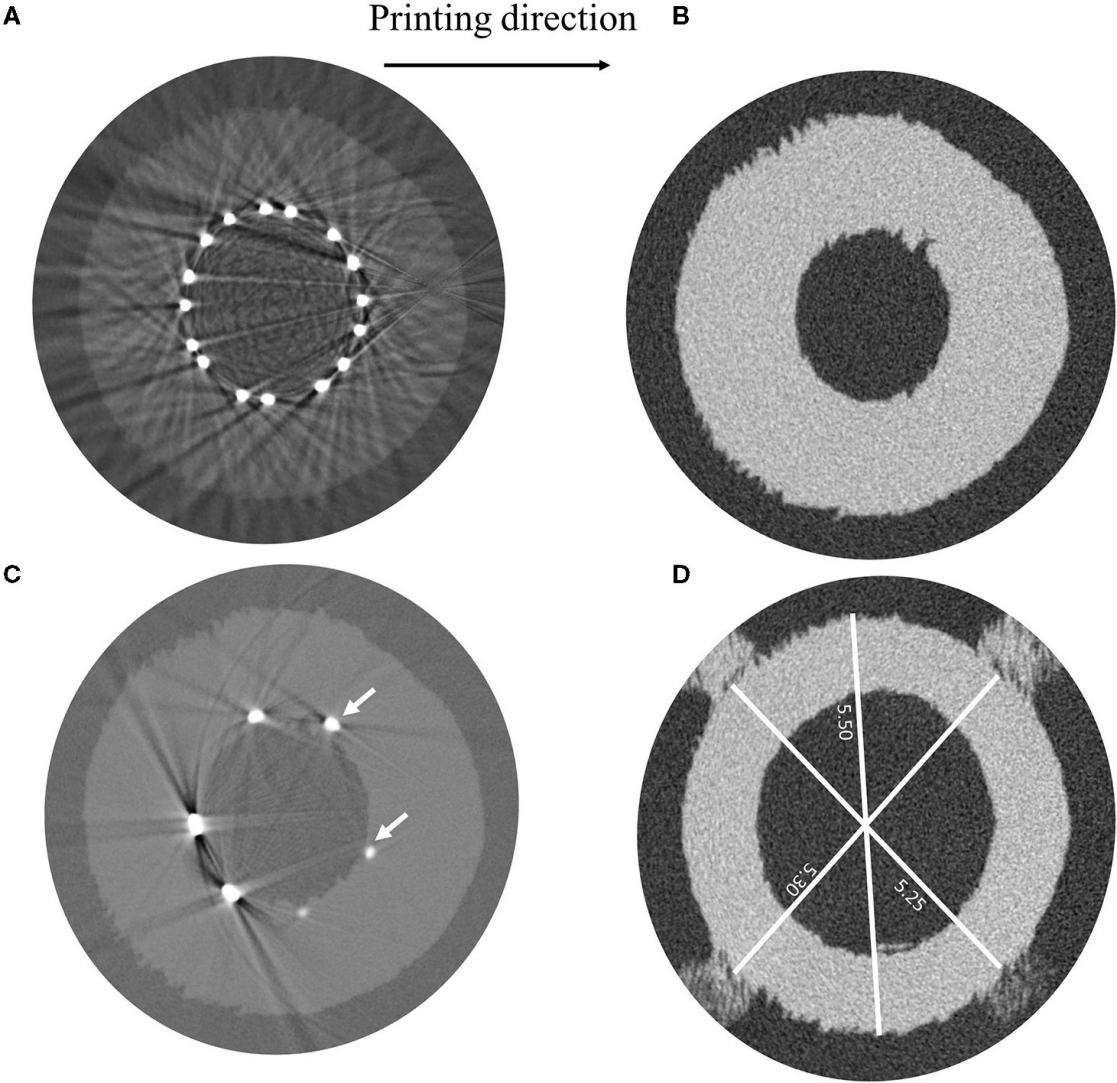
μCT images showing **(A)** irregularities in the vessel geometry ascribable to the 3D-printing process, **(B)** measurements of different vessel diameters, **(C)** light beams reflected by the SYNERGY™ stent during the acquisition preventing the evaluation of the struts' malapposition, and **(D)** detail of struts indenting the vessel wall. Indication about the printing direction is provided.

The μCT images, taken a few days after the test, showed irregularities on the surface of the vessel related to the layer deposition during the 3D printing process (Figure 7A). These peculiarities can be observed along the entire length of the vessel, which also showed an overall ovalization (Figure 7B).

Unfortunately, it was not possible to achieve information regarding the existence of the strut malappositions, which were detected in the model, because of artifacts related to the light beam scattering from the metallic surface of the stent (Figure 7C). Moreover, by looking at those acquisitions showing the initial portion of the stent, where only few struts appear and the beam scattering is reduced, it was possible to ascertain that the stent struts indented the vessel wall (Figure 7D). For this reason, since the vessel lumen was not clearly visible and the value of the outer diameter could be altered by the damage in the vessel, only the final configuration of the stent was investigated, which showed good agreement in terms of the stent outer diameter between the model and the μCT images (Table 3).

**Table 3:** Comparison between the average measure of the stent outer diameter through μCT and numerical simulations after deployment in the idealized vessel design.

**Stent outer diameter (mm)**			
**Marker**	**FEA**	***μ*** **CT (mean value)**	**%Err**
**ID**			
2	3.01	2.98	1.1%
3	2.89	2.80	3.1%
4	2.87	2.76	3.7%

**Figure 8:**
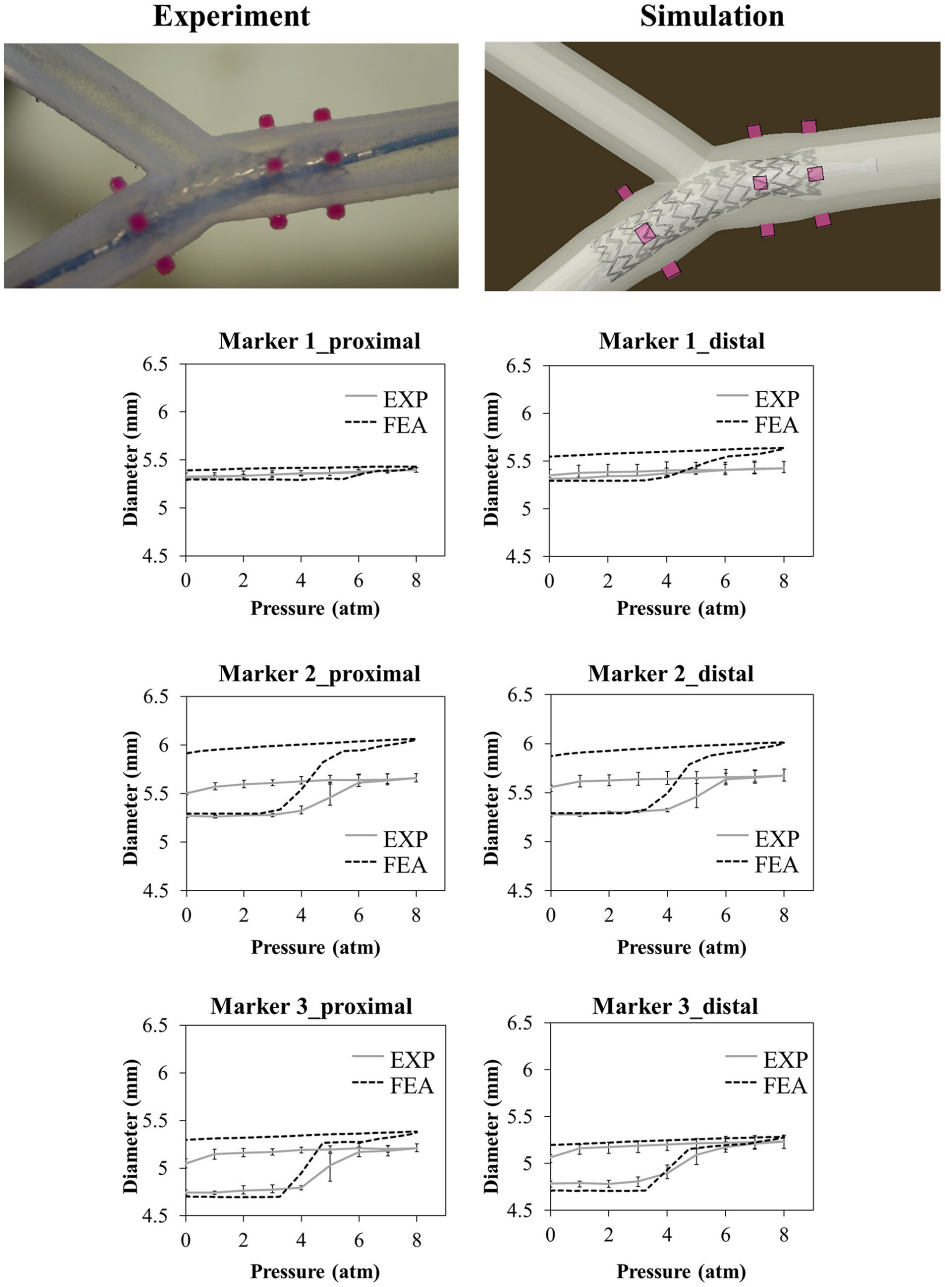
Comparison between experimental and numerical results of the deployments of the SYNERGY™ stent in a bifurcated vessel, taken at Marker 1,2,3 (from proximal to distal). A qualitative comparison between experimental and numerical configuration is reported considering the final instant of the balloon deflation phase.

The results of the deployment into the bifurcated vessel are shown in Figure 8. The model demonstrated a different ability in matching the experimental data at different locations. Table 4 reports the comparison in terms of diameter gain.

**Table 4:** Comparison between the diameter gain obtained in the numerical simulations and experiments involving the idealized and bifurcated vessel design.

	**Vessel diameter gain (%)**			
**Marker**	**FEA**		**EXP (mean value)**	
**ID**	**Proximal**	**Distal**	**Proximal**	**Distal**
	**Idealized Vessel**			
2	3.3%	4.4%	2.7%	3.8%
3	7.8%	8.3%	7.2%	6.9%
4	7.6%	7.8%	6.5%	6.3%
	**Bifurcated Vessel**			
1	1.9%	4.9%	0.2%	0.6%
2	11.7%	11.0%	4.4%	5.3%
3	12.8%	10.6%	6.5%	5.8%

**Figure 9:**
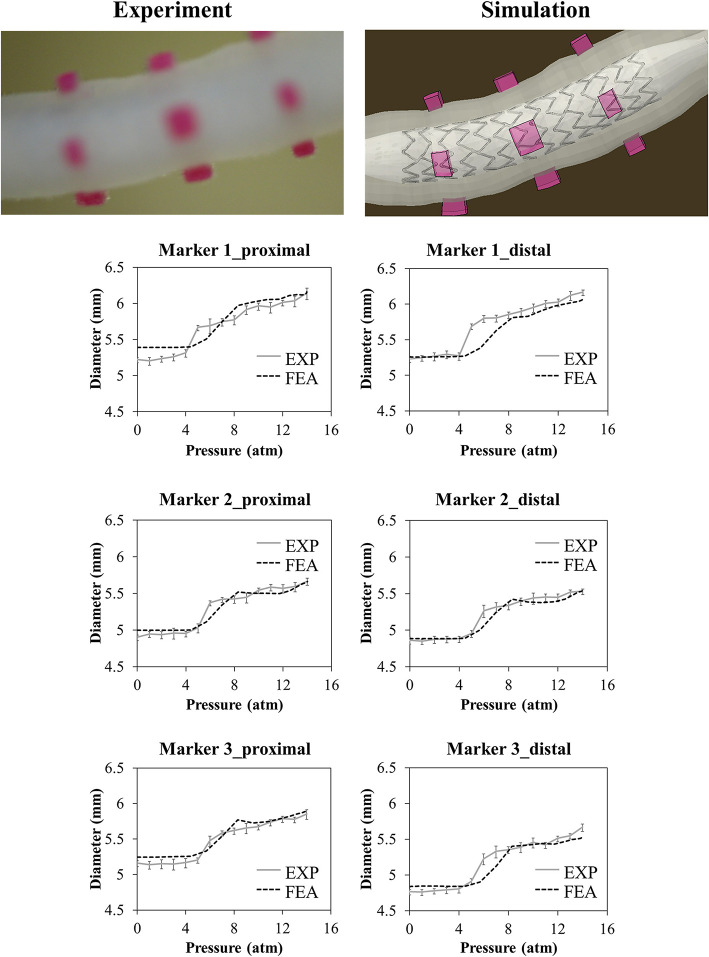
Comparison between experimental and numerical results of the deployment of the SYNERGY™ in a realistic vessel, taken at Marker 1,2,3 (from proximal to distal). Only data regarding the loading phase are reported due to the vessel rupture at deflation. A qualitative comparison between experimental and numerical configuration is reported considering the final instant of the balloon deflation phase.

In the case of the realistic vessel, the pressure-diameter plots of the loading phase are reported in Figure 9. During balloon deflation, the vessel broke in the region close to the curvature (Marker 3), and it was not possible to exploit the data regarding lumen gain.

**Figure 10:**
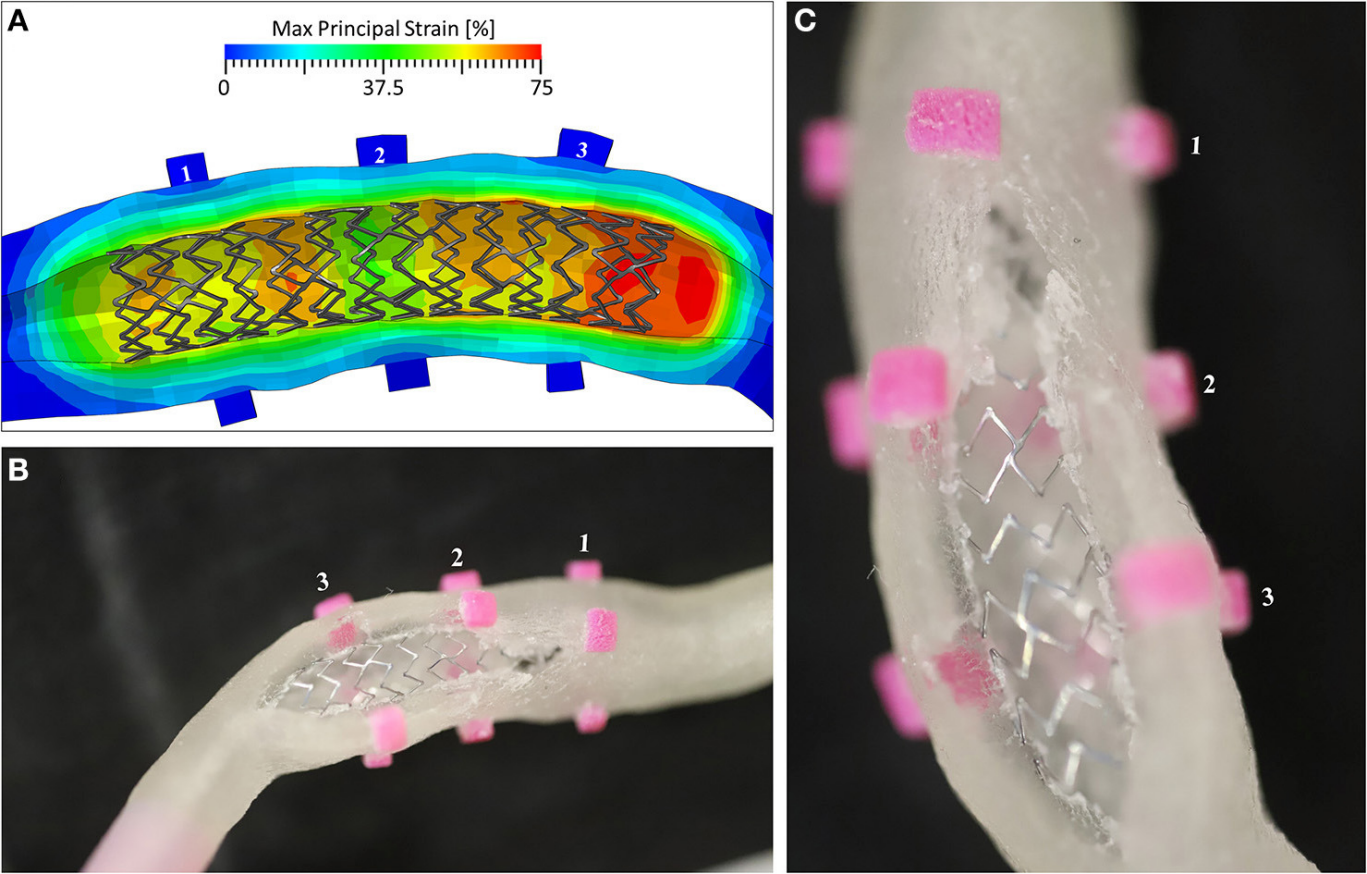
**(A)** The Max Principal Strain plot at the end of the inflation phase, showing peaks of 75% deformation in the area of Marker 3; **(B)** the view of the broken mock vessel with **(C)** a detail of the exposed stent.

To better understand the reasons for the failure, the numerical model was used to evaluate the local strain at the location of the rupture. The results demonstrated that in the zone in which the experimental event took place, the local strain reached a value of 75%, which is compatible with the ultimate limit found in the experimental tests on the ring samples (Figure 10).

## Discussion

This study suggests *in vitro* comparators to validate numerical models of coronary stent deployment to be used in the evaluation of the acute outcome of the procedure. In particular, few experiments involving 3D-printed mock vessels, which could be performed in most research laboratories, are proposed, and indications of the quantities of interest that could be evaluated are given. There are currently numerous examples of literature studies producing elastomeric phantoms exploiting the versatility of PolyJet technology, among which only few are specifically designed for stent implantation (17–19). However, to the best knowledge of the authors, all the reported studies involve deployment sites (e.g., abdominal aorta, thoracic aorta, or intracranial arteries) characterized by wider lumens than coronary arteries and used self-expandable devices were deployed not to reopen a stenotic vessel but to treat an arterial aneurism. This could explain the two major issues faced in this study when these mock vessels were printed to mimic stenotic coronary arteries: on one hand, the very small dimensions of the lumen might add issues in the fabrication because of resolution limits, and on the other hand, the strong interactions between the device and the vessel wall due to the high pressure of the balloon might create strength issues for the printed material.

In this context, the material characterization activity showed mechanical properties similar to those in the manufacturer datasheet (e.g., elongation at break between 220–270%): however, the results of the annular tensile tests showed a significant decrease in the ultimate strain, which could be explained by a major weakness in the bond between consecutive printed layers (out-of-plane direction). This could be recognized as a limitation of the PolyJet technology for this purpose, which has to be acknowledged during the planning of experimental activities to avoid ruptures by layer detachment as in the case of the deployment into the realistic vessel.

Even if PolyJet allows in principle the realization of components characterized by different mechanical properties, the authors decided not to exploit this feature in the differentiation of the arterial layers, namely, the intima, media, and adventitia. Indeed, the final aim of the mock vessels is to mimic global compliance similar to that of coronary arteries, to act as a constraint to the stent expansion due to the balloon inflation. Hence, it was decided to design all the mock vessels characterized by a single material (i.e., Agilus30) whose mechanical properties can be considered as intermediate in terms of stiffness between the plaque and the media/adventitia layers (20, 21). Moreover, the use of multiple materials can create additional issues during stent deployment since the interfaces of different materials may result in lower mechanical strength.

Another important aspect of the phantoms is the dimensional mismatch between the CAD and the diameter measured with high-resolution camera in a temperature-controlled wet environment. Taking measurements with samples immersed in water could indeed introduce some uncertainties: however, since both positive and negative variances were obtained, a scaling effect due to water could be discarded. The maximum difference between CAD and sample was found in the case of the idealized vessel and assessed at 190 μm, which is not negligible since it is in the order of magnitude of the vessel diameter gain (Figure 5). This disagreement had an immediate consequence in the results of the deployment in the idealized vessel, as reported in Figure 5: the main difference between the experimental and numerical curve was due to this dimensional mismatch, causing a shift of the numerical results of the vessel diameter. A solution to this limitation, which is demanding in terms of efforts and resources, would be obtained by preparing the finite element model of the vessel starting from a CAD obtained through an imaging reconstruction from the real printed samples.

The choice of assessing the outputs of *in vitro* deployments at different time steps resulted in being successful in supporting the interpretation of the results, allowing to recognize limits of the mock vessels; on the other hand, considering the values of the diameter only at maximum inflation or at the end of the deployment would make more difficult the evaluation of the model ability to represent the reality of interest.

Given the limited transparency of the mock vessels, the variation of the outer diameter was chosen as a comparator. This would provide a quantitative but indirect index of the role played by the stent in lumen enlargement. This choice simplified the experimental setup by avoiding the use of OCT catheters or other more sophisticated imaging techniques that could not be feasible in most research laboratories. However, it introduced few aspects to be aware of, in particular, the fact that the measure of the outer diameter of the vessel was related to the lumen gain, which was the real quantity of interest, through the wall thickness: this, in turn, was affected by the value of the Poisson's ratio, here assumed close to 0.5, and the possible indentation of the struts.

The outer diameter was proven to be a suitable choice, especially in the case of the idealized vessel, where the experiment was able to indicate the stent apposition to the lumen (i.e., the movement of Markers 2, 3, and 4) and possible malapposition (i.e., Marker 1). These results were representative of the device compliance chart and were also observed through simulation.

The analysis of data provided by the μCT images gave the possibility of discussing the issue linked to the uncertainties affecting such measurements: in fact, μCT images are taken under different environmental conditions than the tests, namely, in the air at room temperature; in this study the samples were transferred to a different laboratory, meaning that the acquisitions dated back to few days after the deployment. While it may be possible to perform the scans a few minutes after the test and include an insulated bath to maintain the sample hydrated, it would become much more challenging to actively control the temperature of the bath, which means that it would be likely that the temperature would change over the timeframe of the scan (which can last several hours), which may not be ideal with rate and temperature-dependent materials.

μCT images performed on the extremities of the idealized vessel (that can be considered as unaffected by the stenting procedure) showed differences between the CAD and the real sample (Figure 7B): the measure of the diameter in correspondence of markers was lower than the nominal value of 5.40 mm, between 5.25 and 5.3 mm, while the maximum value was found considering the diameter parallel to the printing plane (5.5 mm). The samples are introduced in the μCT machine in vertical position and, to avoid undesired deformations of the samples due to gravity, they should be equipped with supports included on the outer surface of the vessels, ideally in a location that is not in proximity to any deployed stents to ensure that the support would not provide any mechanical reinforcement to the vessel in that region. The supports could either be part of the initial 3D print job, such that the vessel is all one part, or a separate polymer-based support could be printed, which would house the vessel during scanning. This solution would improve the correspondence between μCT acquisition and model outputs, lowering measurement errors due to sample positioning.

Unfortunately, in the case of a metallic stent, artifacts due to beam scattering precluded clear visualization of the lumen and, hence, the quantification and location of the malapposed struts (Figure 7C); this technique would be useful in the case of polymeric stents, where such artifacts are not present because of similar densities of constituent materials. On the other hand, while other techniques could be successful with metallic parts (22), these were unavailable to the authors at the time of the study, and the use of μCT images to assess the lumen was not as quantitative as expected.

A closer investigation of these slices, characterized by limited scattering, showed a clear strut penetration inside the vessel wall (Figure 7D). This could be explained by the local failure of the material due to the prolonged load applied by the stent struts. This called into question the choice of the Agilus30 as the constituent material for the vessels.

A possible improvement in the direct acquisition of the information regarding the lumen during the test would be provided by the use of a transparent material for the fabrication mock vessels, which at the same time should maintain proper mechanical properties and printability.

The choice of a more complex mock vessel, characterized by the presence of simplified bifurcation and calcification, theoretically adds value to the validation, increasing model reliability in answering the question of interest. Unfortunately, the results in Figure 8 show that the model overestimated the experimental value of the diameter of the vessel, especially at Marker 2 that is in proximity with the calcification. Since two different materials were involved in the 3D printing of this small area, possible reasons may explain this mismatch: (i) some limitation in its realization could arise leading to a final product that could be different from the expected (and simulated) one in terms of geometrical features and material properties; and (ii) in the experiment, the stent struts possibly had indented the Agilus30 layer covering the calcification (about 350 μm from the CAD measure): this could have led to a lower movement of the outer diameter compared with the one obtained through the model.

On the other hand, the realistic vessel represented an interesting solution, providing a high degree of similarity to the *in vivo* application and, hence, being a good choice for addressing the question of interest.

In conclusion, the use of 3D-printed mock vessels for comparator study within the validation process for assessing model credibility of coronary stent deployment, according to V&V 40, requires the following steps:

1. Accurate choice of material for mock vessels. It implies the definition of an experimental campaign for mechanical characterization. Following the experience of this study, it is recommended to select a testing configuration that mimics material loading under working conditions. Indeed, because of a lower bonding force between consecutive layers, the annular tensile test showed a significant reduction in the ultimate strength when compared with the tensile test on dog-bone samples;
2. Selection of different vessel designs to prepare CADs for a study. The overall mechanical properties have to be in the range of interest, namely, similar to the radial strength of arteries. Especially for more realistic designs, it is important to equip the vessel with markers that could support the comparison between numerical and experimental outputs at univocal locations;
3. Quantifications of uncertainties of the manufacturing process. Once printed, checking of the vessel morphology should be done in terms of outer diameter at different locations. This could be performed using optical systems, such as a high-resolution camera, which provide indications of those samples being much different from the nominal CAD. Since geometrical uncertainties are expected, it would be recommended to print several copies of each design to exploit only those characterized by good printing accuracy;
4. Fine-tuning of the experimental setup for deployment tests. For all polymeric and metallic stents, tests should be conducted in a temperature-controlled environment with the samples immersed in water. An optical system should be used to monitor the displacement of the outer diameter of the vessel in correspondence with the rigid markers;
5. Last, the numerical model of stent deployment has to be prepared. Here, a simplified but effective strategy for simulation is presented. Comparison between model outputs and experimental results concludes the validation pathway.

At different stages of the study, even if it is not common to have access to such measurements, μCT images could represent a useful tool allowing the evaluation of the vessel lumen and stent implanted configuration.

As shown in this study, following the steps summarized here may give promising results. However, the current limitations of printing techniques and materials must be overcome before this approach can be applied extensively. Recent studies (23) that proposed technological upgrades in the field of printable materials seem to indicate a possible future direction in developing a reliable comparator.

## Data Availability Statement

The original contributions presented in the study are included in the article/supplementary material, further inquiries can be directed to the corresponding author.

## Author Contributions

FB and LA were responsible for writing the manuscript. FB was responsible for results visualization. LA and GPo performed the experimental and numerical activities involving stent deployment. CF and TV performed the microCT scans. FM, LP, and GPe supervised the activities. LP and GPe were responsible for the work conceptualization. All authors contributed to the article and approved the submitted version.

## Conflict of Interest

The authors declare that the research was conducted in the absence of any commercial or financial relationships that could be construed as a potential conflict of interest.

## Publisher's Note

All claims expressed in this article are solely those of the authors and do not necessarily represent those of their affiliated organizations, or those of the publisher, the editors and the reviewers. Any product that may be evaluated in this article, or claim that may be made by its manufacturer, is not guaranteed or endorsed by the publisher.
